# *Nippostrongylus brasiliensis* infection leads to impaired reference memory and myeloid cell interference

**DOI:** 10.1038/s41598-018-20770-x

**Published:** 2018-02-13

**Authors:** T. M. H. Bogale, K. S. De Gouveia, L. Cruywagen, N. Makena, F. Booley, O. Tamgue, F. Brombacher

**Affiliations:** 1 International Centre for Genetic Engineering and Biotechnology (ICGEB), Cape Town component, Cape Town, South Africa; 2 Division of Immunology, Institute of Infectious Disease and Molecular Medicine (IDM), Health Science Faculty, University of Cape Town, Cape Town, 7925 South Africa; 3 South Africa Medical Research Council (SAMRC), Cape Town, South Africa; 4 Department of Human Biology, University of Cape Town, Observatory, Cape Town, 7925 South Africa

**Keywords:** Parasitic infection, Spatial memory

## Abstract

Hookworm infection is endemic in developing countries, leading to poor cognitive function—among other disruptions. In this study, the effects of *Nippostrongylus brasiliensis* infection (a murine model of *Necator Americanus*) on cognitive function were investigated. Though impaired cognition has been extensively reported, the exact domain of cognition affected is still unknown, hence requiring investigation. The objective of this study was to identify possible cognitive changes during *Nippostrongylus brasiliensis* infection in mice, using the Morris water maze. Here, we show for the first time that mice infected with *Nippostrongylus brasiliensis* were able to learn the Morris water maze task, but demonstrated impaired reference memory. Anxiety measured by thigmotaxis in the maze, did not play a role for the observed cognitive impairment. Of further interest, an increase in the number of hippocampal macrophages and microglia with training and/or infection suggested a significant role of these cell types during spatial learning. Together, these experimental mouse studies suggest that helminth infections do have an impact on cognition. Further experimental animal studies on cognition and infection might open new approaches for a better understanding and impact of pathogen infections.

## Introduction

One of the major immune responses to helminth infection include CD4 T-helper 2 (Th2) activity^1,2^. Indeed, effects of helminth infection on cognitive function have been a debatable topic for many years. Various studies have linked helminth infection to cognitive impairment^3–5^. For example, studies in helminth infected children showed correlation by reduced cognitive scores than non-infected children. This lead to the general consensus that improvement in cognitive function might occur after helminth treatment^6,7^. Indeed, deworming in individuals infected with hookworm and/or other helminthes was reported to result in improvement of cognitive function^6^. Unfortunately, re-infection is common in endemic areas, even with regular deworming^8^, resulting in possible long-lasting observed cognitive dysfunction. Although hookworm infection is known to occur in childhood, it is reported that both frequency and intensity of infection remain in adulthood^9^. Hence, there is a lack in the knowledge of which cognitive domains—if any— are affected, along with mechanisms involved in the process of learning and memory during helminth infection. Our results suggest impairment of reference memory, but not learning.

At baseline and/or low physiological levels, pro-inflammatory cytokines are known to be essential for effective cognitive functioning but are detrimental at high, pathological concentrations (e.g. during infection), where they may play a role in impaired learning and memory^10–14^. On the other hand, anti-inflammatory cytokines IL-4 and IL-13—both up-regulated during *Nippostrongylus brasiliensis (N. brasiliensis)*^15^ infection, may influence cognition^16,17^. In addition, administration of bacterial toxin has been shown to result in both impairment of memory and increased anxiety^18^, indicating a possible association between anxiety and cognitive function.

The cognitive domains of interest in this particular study include learning and reference memory. An important component of learning is that it is not stored because it is relevant to the activity at hand, while reference memory is a form of hippocampal-dependent long-term memory that, unlike learning, becomes more stable over time, as it requires consolidation to occur^19^. We therefore investigated hippocampal activity to better understand its contributions towards learning and memory^20–23^. Here, we describe how *N. brasiliensis* infection influences the cognitive domains of learning and memory in an experimental mouse model to better understand the mechanism leading to the reported decline in cognition during infection in human^3–5^.

## Results

### *N. brasiliensis* infection induces hypercontractile responses to acetylcholine

**Figure 1:**
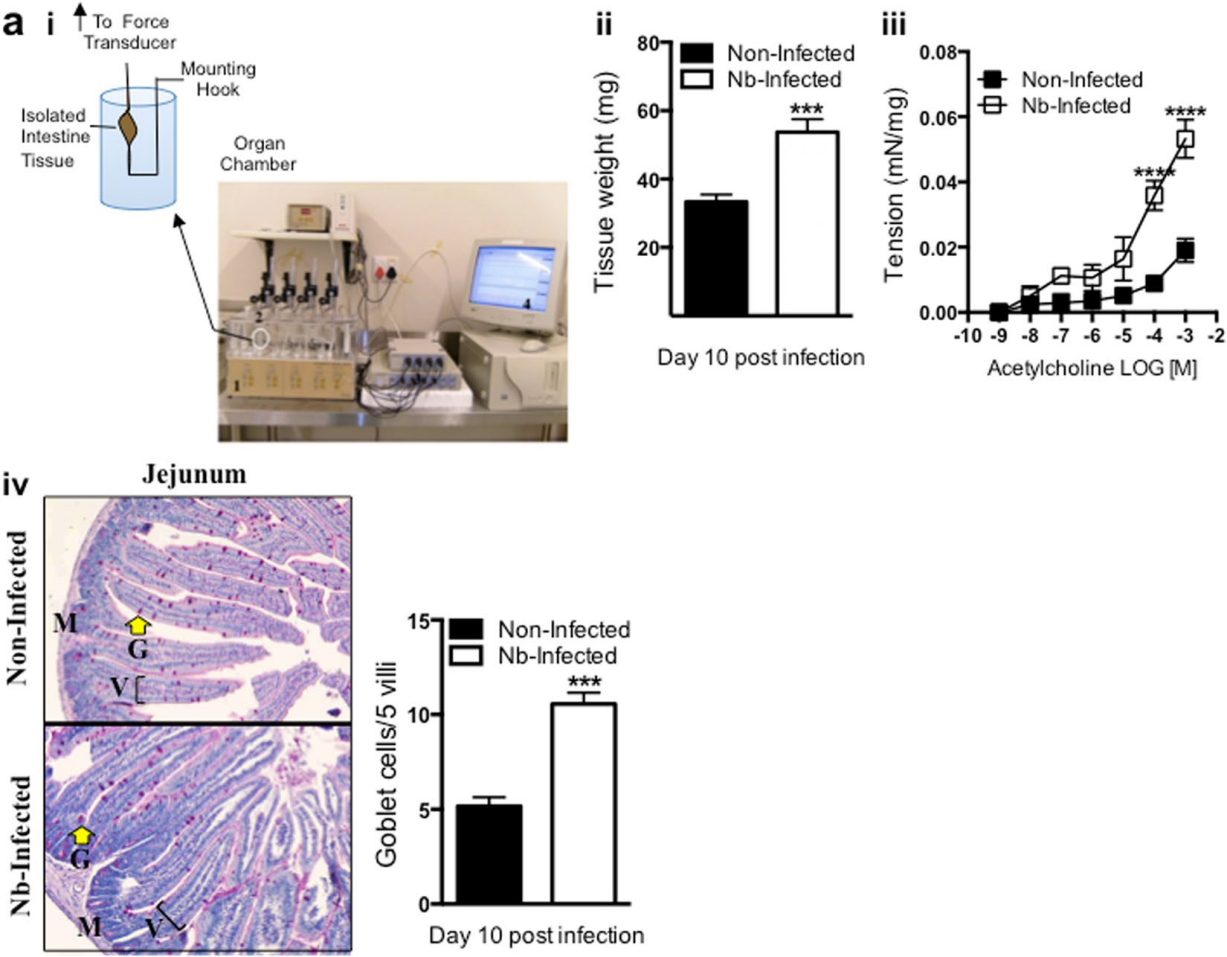
A measure of *N. brasiliensis* infection: On day 10 post infection, approximately 1 cm jejunum segments were removed from the small intestine of both non-infected and *N. brasiliensis* infected Wild-Type mice. (**a**) A pictorial representation of the water-jacketed organ bath (Panlab, Spain) used to measure smooth muscle contractile responses is shown, connected to transducers and the PowerLab^TM^ system (ADInstruments, Australia) [Picture taken by Dr. T.M.H. Bogale, 2010]. This feeds and translates the signal to a computer for measuring tissue isometric tensions (i). Tissues were weighed on an analytical scale (ii) showing an increase in tissue weight by *N. brasiliensis* mice, before stimulating with varying concentrations of ACh −9 to −3 LOG [M] to determine isometric contractile responses (iii) showing an increase in tissue hypercontractility of infected intestine. Mucus producing goblet cells were determined by PAS reagent at day 10 post infection on jejunum sections of non-infected and *N.brasiliensis* infected Wild-Type mice. Goblet cell hyperplasia was determined by counting the total number of enlarged goblet cells per 5 villi from 5 tissue sections isolated from 5 individual mice to a total of 25 villi. Mucus-producing goblet cells were visualized using PAS reagent staining (iv) demonstrating hyperplasia by infected intestine tissue [100X magnification]. M: Muscularis, G: Goblet cells [arrow heads], V: Villus [bordered lines]. Results are the mean ± SEM (n = 4 mice/group Unpaired t-test ***P < 0.001 for tissue weight; n = 10 mice/group Two-way repeated measures ANOVA with Bonferroni post-hoc test used for individual time-point comparisons ****P < 0.0001 for hypercontractility; n = 5 mice/group Unpaired t-test ***P < 0.001 for goblet cells. Results are a representation of three independent experiments.

Wild-type mice of Balb/c background were infected with ~750 L3 infective stage larvae and tissue samples were collected at day 10 post infection. *N. brasiliensis* infected mice showed an increase in tissue weight (Fig. 1a.ii), and increased hypercontractile responses to acetylcholine challenge compared to non-infected controls (Fig. 1a.iii). Goblet cell hyperplasia was determined from jejunal sections (Fig. 1a.iv) by quantifying the total number of enlarged mucus producing goblet cells stained with periodic acid Schiff’s reagent (PAS) shown by arrow heads, demonstrating increased thickness of the muscle layer of *N. brasiliensis* infected mice with evident goblet cell hyperplasia. These results taken together confirm successful *N. brasiliensis* infection, as previously shown^24^.

### *N. brasiliensis* infection impairs reference memory, but not learning

**Figure 2:**
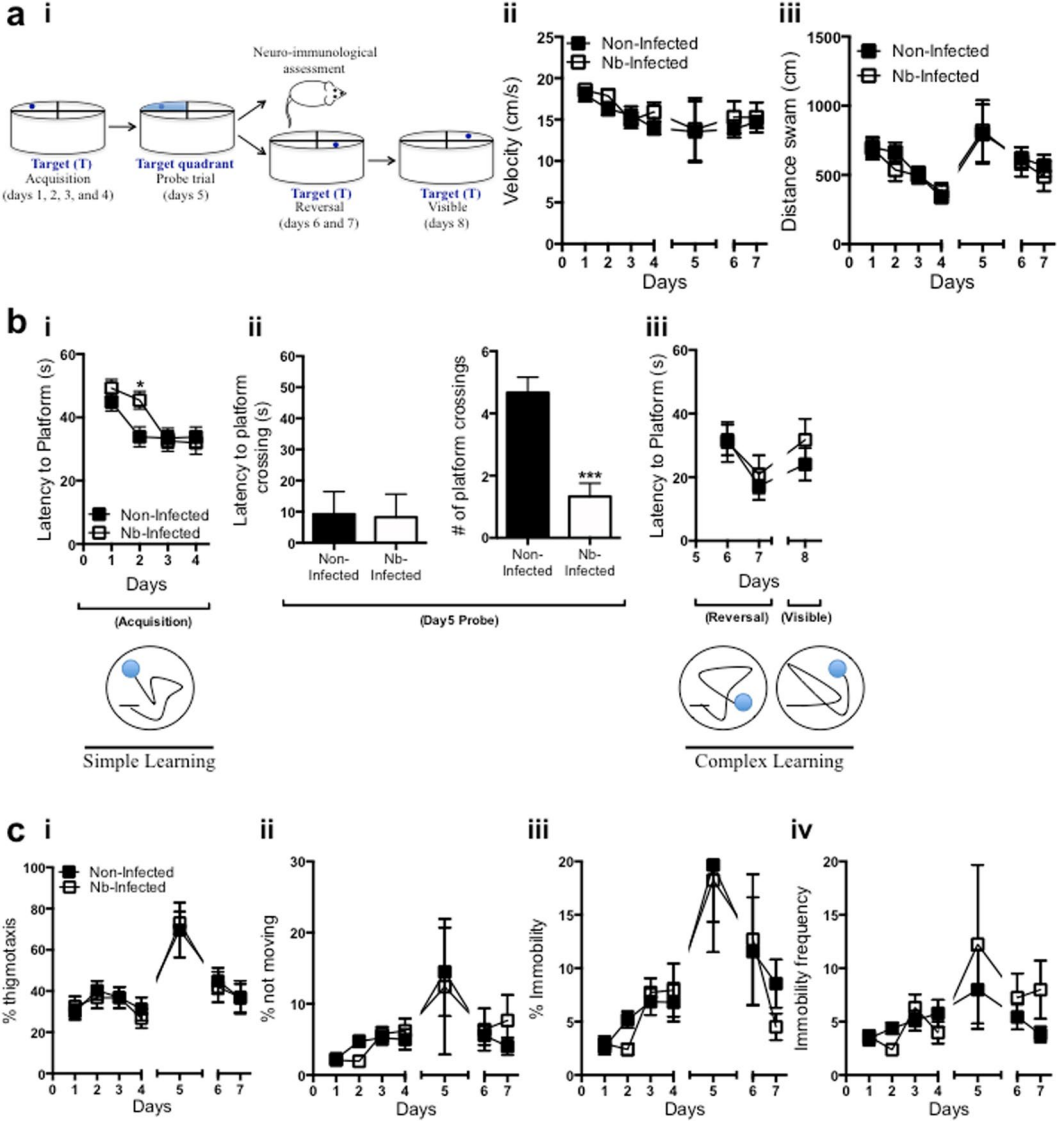
Impaired reference memory but not working memory induced by *N. brasiliensis* infection is not influenced by speed, distance swam, or anxiety in the MWM. (**a**) A pictorial representation of the Morris water maze used to assess hippocampal dependent spatial learning and memory is shown (i) [Picture, including mouse, drawn by Dr. T.M.H. Bogale, 2017]. Balb/c mice were infected with ~750 L3 *N. brasiliensis* and trained in the MWM with Velocity (ii) and distance swam (iii) measured as controls for any differences seen in cognitive measurements. (**b**) Mice were then trained in the MWM and latency to platform measured during acquisition (i) showing no differences across treatments during the simple learning phase of the task. A probe trial was performed on day 5 to measure latency to platform crossings and number of platform crossings (ii) with no differences between treatment conditions. Latency to platform was measured again during reversal and visible phases of the task (iii) with no differences across treatment conditions during these complex learning phases of the task. (**c**) Mice were assessed in the MWM and % thigmotaxis (i), % not moving (ii), % immobility (iii) and immobility frequency (iv) were measured as indicators of anxiety, and no differences were observed across treatment conditions. Results are the mean ± SEM (n = 18 mice/group; Unpaired t-test ***P < 0.001; Two-way repeated measures ANOVA *P < 0.05, with Bonferroni post-hoc test). Figures are a representation of 7 independent experiments.

In order to rule out performance as a reason for differences across treatment groups, data from both *N. brasiliensis* infected and non-infected mice were analyzed for speed (velocity) and distance swam. Infected and non-infected mice demonstrated similar performances (Fig. 2a.i–iii). This result is an important control to rule out the possibility that infection with *N. brasiliensis* influences behavioral factors, besides cognition. Mice were then trained in the MWM and latency to platform measured during acquisition (Fig. 2b.i,iii). Statistically significant differences were not observed during learning, suggesting that *N. brasiliensis* infection does not negatively impact on learning, whether “simple” or “complex”. However, on a test of reference memory during the probe trial phase of the task, *N. brasiliensis* infected mice showed fewer platform crossings compared to non-infected mice (Fig. 2b.ii), demonstrating impairment of this domain of cognitive function. Following the observed result of impaired reference memory, but not learning, we hypothesized anxiety as a possible cause of interference in memory consolidation, and was therefore investigated. Anxiety was determined by duration swimming within 10 cm of the MWM edges, subject freezing in response to stimuli, immobility (indicating no limb movement), as well as not moving (where there is no spatial displacement by the subject). Results from these behavioral markers demonstrated that anxiety does not play a role in impaired reference memory, but rather, attenuated reference memory is directly due to *N. brasiliensis* infection as demonstrated by no statistical differences between *N. brasiliensis* and non-infected mice (Fig. 2c.i–iv).

Taken together, these results suggest that infection by *N. brasiliensis* is not detrimental for learning, but rather for reference memory, suggesting that memory consolidation could be disrupted in this model, and that this disruption is not due to anxiety.

### MWM training and *N. brasiliensis* infection influence population dynamics of macrophages and microglia within the Hippocampus

**Figure 3:**
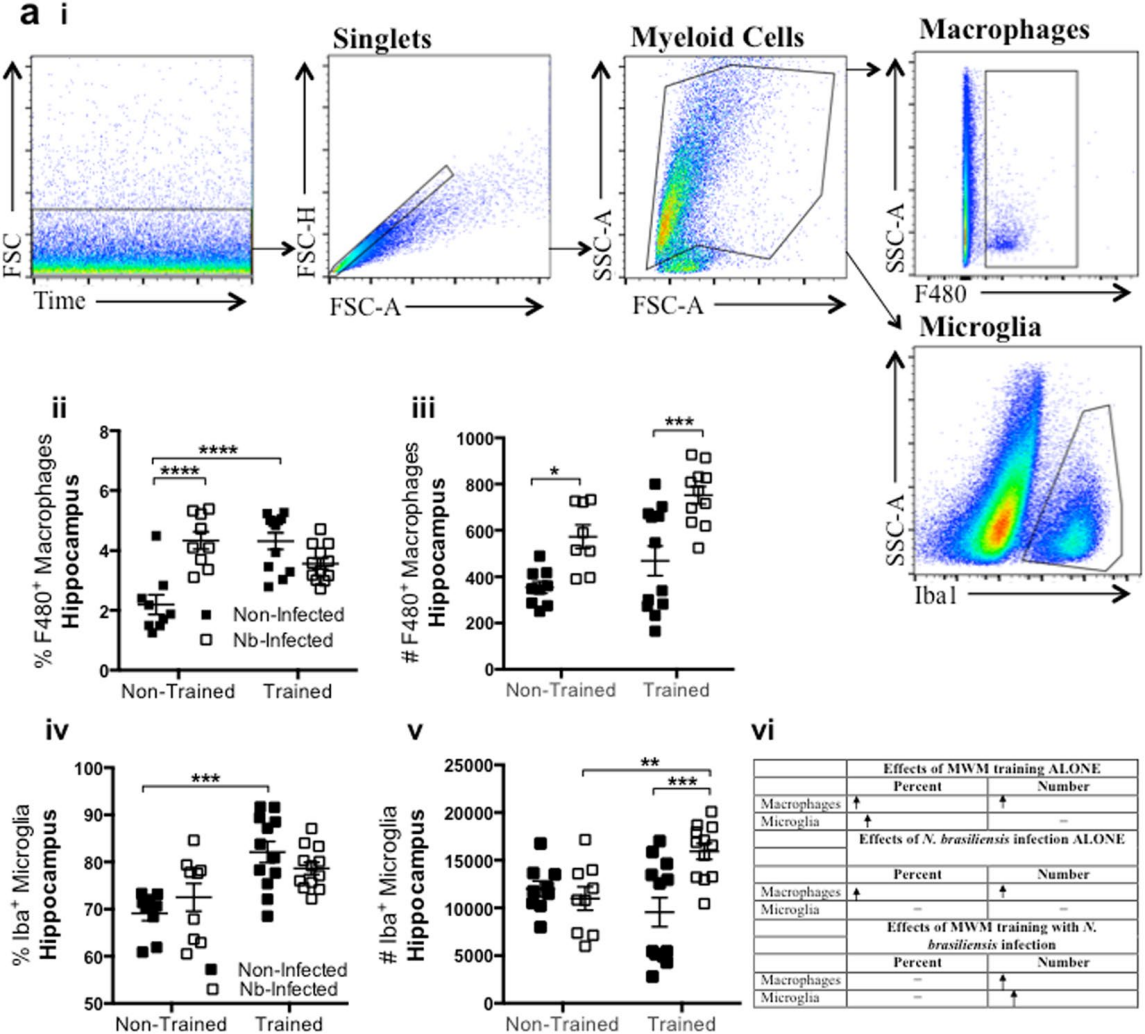
*N. brasiliensis* infection alters hippocampus macrophages and microglia to influence cognitive function. (**a**) Gating strategy for identifying F480^+^ macrophages and Iba1^+^ microglia is shown. Single cell suspensions from the hippocampus of *N. brasiliensis* infected and non-infected mice were examined by flow cytometry for F480^+^ macrophages (ii, iii) showing an increase in macrophages by MWM training as well as by infection, and for Iba1^+^ microglia (iv, v) demonstrating statistically significant increase of microglia by MWM training, but not infection following spatial learning task. A summary of results is shown (vi) as the mean ± SEM (n = 4 mice/group; Two-way ANOVA *P < 0.05, **P < 0.01, ***P < 0.001, **** p < 0.0001 with Bonferroni post-hoc test). Figures are a representation of 3 pooled independent experiments.

In an attempt to better understand neuro-immunological mechanisms underlying the observed detrimental effects of *N. brasiliensis* on reference memory, macrophage and microglia responses in the hippocampus of *N. brasiliensis* infected and non-infected mice were examined before and after MWM training. Hippocampal cells stained with an immunofluorescent antibody against macrophages (F480) and microglia (Iba1) were identified by FACS analyses, with a series of gates to allow identification of F480^+^ and Iba1^+^ cells (Fig. 3a.i). Interestingly, MWM non-trained but infected mice had an increased percentage (Fig. 3a.ii) and numbers (Fig. 3a.iii) of F480 positive macrophages in the Hippocampus (Fig. 3a.iii). Once trained, non-infected mice also increased the percentage of F480 positive macrophages, similar to the infected mice (Fig. 3a.ii). In absolute numbers, non-infected mice showed similar F480 positive macrophage numbers within the hippocampus, irrespective of training (Fig. 3a.iii), whereas infected mice increased the number of F480 positive macrophages in the hippocampus (Fig. 3a.iii). Non-infected Iba positive microglia showed similar percentage and numbers, whereas infected mice increased the percentage and numbers of Iba positive microglia (Fig. 3a.iii). These results suggest that *N. brasiliensis* infection as well as MWM training in mice may have effects on the population dynamics of F480 positive macrophages and Iba positive microglia within the hippocampus.

## Discussion

Because peripheral products and substances are able to infiltrate the central nervous system by various means^25,26^, the effects of *N. brasiliensis* infection were investigated within the brain to assess possible influences on cognitive function. Although helminth infection is known to cause cognitive impairments in children, the exact domain and mechanism of cognitive impairment are unknown^3–5^. In this study, we demonstrate a central role for *N. brasiliensis* infection in the regulation of cognitive function. We show for the first time that *N. brasiliensis* infection leads to partial impairment of cognitive function, as demonstrated by disrupted reference memory, but not learning. We further found that *N. brasiliensis* infection leads to accumulation of macrophages in the hippocampus.

Both *N. brasiliensis* infected and non-infected mice displayed similar latencies to the platform during “simple” (acquisition) and “complex” (reversal) learning tasks (Fig. 2b.i,iii), with fewer platform crossings by *N. brasiliensis* infected mice during a measure of reference memory (Fig. 2b.ii). This result indicated that even though *N. brasiliensis* infection impairs cognition, learning is protected, while reference memory seems to be attenuated by a mechanism that is yet to be determined. Although learning was not affected during *N. brasiliensis* infection, there was a constant increase in latency to platform by *N. brasiliensis* infected mice on day 2 of MWM training (Fig. 2b.i). *N. brasiliensis* penetrates the skin to migrate to lungs by means of the vasculature where they reach airspaces to be coughed up and swallowed in 3–4 days^27^. As a result heavy mucus production in the lungs at this time point of infection^28^ may have interfered with “normal” breathing, which could have had an influence on task performance and outcome.

While previous studies did not determine the cognitive domains affected by hookworm infection^3,29^, it is investigated and indicated here for the first time.

Having determined that *N. brasiliensis* infection impairs reference memory, but not learning, the role of anxiety in impaired cognition of *N. brasiliensis* infected mice was investigated. Anxiety is known to influence cognition both positively and negatively^30^. Because the MWM task is a novel environment, anxiety levels were determined to further characterize the cognitive impairment observed. Anxiety levels, as determined by thigmotaxis, including freezing, not moving, and immobility, were similar between *N. brasiliensis* infected and non-infected mice (Fig. 2c), ruling out possible anxiety as a factor contributing to the impairment of reference memory observed during infection. Currently, there is no evidence that anxiety may be associated with decreased neurogenesis in the hippocampus, however anti-anxiety medication does increase neurogenesis in rodents^31^. Indeed, anxiety-related stress, as opposed to fear-related stress may cause attenuated learning^32^. To further substantiate our findings of impaired reference memory, the role of cells in the hippocampus was investigated. Evidence for hippocampal microglia and macrophages influencing cognition through enhancement/inhibition of neurogenesis^33,34^, incited the idea that these cells could be involved in the process of leaning and memory during *N. brasiliensis* infection. In addition, blood-borne macrophage recruitment to the brain has been shown to have detrimental effects on cognitive ability, although we do not show evidence of peripheral macrophages invading the central nervous system^34^. In our study, macrophage numbers were determined within the hippocampus. Of interest, MWM training with or without *N. brasiliensis* infected lead to an increase in macrophages. Moreover, *N. brasiliensis* infection alone lead also to an increase in macrophages, which may suggest the importance of macrophages in cognitive function, as well as defense from *N. brasiliensis* infection. Due to the impairment of reference memory in this model, it is likely that macrophages might be interfering with normal cognitive functioning by producing high levels of pro-inflammatory cytokines in the brain^10,35–38^, while anti-inflammatory cytokines IL-4 and IL-13 would support effective learning and memory^16,17,39^. Although macrophages were not depleted as a means to determine their role in cognitive function following *N. brasiliensis* infection, it has been reported that immune deficient mice injected with alternatively activated macrophages demonstrated beneficial effects on learning and memory of the MWM task^39^. This result substantiates the observations in our study, where we show a positive correlation between macrophages and learning following MWM training or *N. brasiliensis* infection. Because learning was not impaired, it is likely that immune products by macrophages are only detrimental to the cognitive domain of memory formation.

Microglia, which are residential brain macrophages, are known to support neurogenesis in the hippocampus, which is essential for cognition^33,40,41^. However, if these microglia take on a classical inflammatory phenotype (M1), they are instead detrimental to neurogenesis and cognition^42,43^. Therefore, microglial numbers were determined in the hippocampus in order to determine their role in spatial learning following *N. brasiliensis* infection. While training alone and MWM training of *N. brasiliensis* infected mice lead to an increase in microglia, mice that were infected demonstrated no changes, suggesting their role is geared to support cognition, but not to fight *N. brasiliensis* infection. This result also suggests that infection hampers the accumulation and in turn effectiveness of microglia, which is possibly of significance in memory formation. In this case, microglia are able to support reference memory, but not learning.

Taken together, these results showed that increased numbers of macrophages and microglia in the hippocampus of *N. brasiliensis* infected mice might interfere with optimal reference memory in a MWM spatial learning task.

In summary, we revealed that *N. brasiliensis* infection reduces spatial reference memory, independent of anxiety induced by the novel environment of the MWM. Moreover, expansion of microglial and macrophage populations in the hippocampus was associated with a reduction in reference memory.

## Methods

### Animals

Inbred 6–8 week old wild-type Balb/c mice were obtained from the University of Cape Town specific-pathogen-free animal facility and kept in individually ventilated cages. All animals were housed in temperature (21–22 °C) controlled rooms, maintained on a 12-h light/dark cycle and age matched in each experiment, with free access to food and water. Animal protocols were approved by the independent Animal Ethics Research committee at the University of Cape Town, approval number 012/018, and all methods were performed in accordance with the relevant guidelines and regulations.

### *N. brasiliensis* Life Cycle and Larvae Infection

*N. brasiliensis* was passaged through 8 week old Wistar rats by inoculating them with ~5000 3rd stage larvae (L3) per rat in 0.65% saline (a kind gift from Klaus Erb, Wurzburg, Germany). Rats were injected subcutaneously with live L3 in the neck using 18 G needles (Strecan®, B. Braun, Melsungen). At days 6, 7, and 8 post infection (p.i.), faecal pellets were collected in 250 μg/ml Amphotericin B fungizone (Lot# 3079308, Invitrogen Corporation) treated dH2O. The emulsified faecal pellets and Proanalysi charcoal (Merk, Darmstadt) were then mixed to a paste and placed on moist Whatman® filter papers, where eggs were allowed to hatch, and larvae subsequently migrate to the edge of the filter paper. L3 were washed off the edge of the filter paper using 0.9% NaCl and counted under a dissection microscope (Nikon Eclipse) as 5 × 10 μl samples. After counting, the worms were allowed to settle at the bottom of the tube for 30 min. NaCl was pipetted out and larvae re-suspended in 0.9% NaCl at a final concentration of 3750 worms/ml (750 worms/200 μl). Mice were infected subcutaneously on the dorsal aspect of the neck with ~750 *N. brasiliensis* L3 using a 21 G needle (Strecan®, B. Braun, Melsungen). Daily monitoring of sickness behavior^44^ was performed in this study, with mice showing no signs of lethargy, reduced grooming, or loss of appetite. *N. brasiliensis* infected rodents may display symptoms of sickness in a dose dependent manner, whereby “heavy” doses exceeding 2000 larvae induce illness^45^. A dose of ~750 larvae^46^ was used in this study to induce a Th2 response that is characterized by mucus production in the lungs with no signs of sickness behavior.

### Morris water maze

Cognitive function was investigated in both non-infected and infected mice (n = 18) from day one post-infection using the Morris water maze (MWM) over a period of 8 days. Mice were given four trials per day (starting at different locations North, East, South and West) for four consecutive days to locate a plexiglass circular platform (10 cm in diameter), which was placed approximately 0.5 cm below water level in an open circular 123 cm diameter MWM. All MWM testing was performed between 9 am and 3 pm during the lights-on phase. The water and room temperature were kept constant between 21–22 °C and 20–24 °C respectively^17,18^. Balb/c mice used in this study have poor vision^47^ and cannot fully see shapes and objects, although they can distinguish light from darkness^48^, therefore a light source was placed behind the Morris water maze, parallel to the platform position to serve as an external distal cue, where lux by the platform quadrant measured 1 and other quadrants had a lux of 0^17^. During the acquisition phase of the task, each mouse was allowed a maximum of 60 seconds to locate and climb onto the platform. Once the mouse had located the platform, it was given approximately 10 seconds to remain on the platform before returning it to its home cage with infrared heat lamps. Mice that failed to locate the platform within 60 seconds were gently guided to the platform and allowed to acclimatize for 10 seconds before returning to the home cage^16^. On day 5, a probe trial was performed with the platform removed in order to test reference memory^23^. Each mouse was given a maximum of 60 seconds in the Morris water maze. On days 6 and 7, the platform was placed in the quadrant opposite the original training quadrant, and mice retrained for four sessions each day (reversal phase). On day 8, mice were introduced to the pool with a visible platform in a different quadrant, placed approximately 0.5 cm above water level (visible phase). Latency to platform was used as a measure of learning and the probe trial as a measure of reference memory, while thigmotaxis, the tendency of mice to swim within 10 cm of the edges of the maze^49^, was used to assess anxiety^50^. Immobility indicates complete lack of movement, including that of limbs and grooming, while not moving indicates lack of spatial displacement. Data was recorded using the EthoVision® XT 8 automated tracking system (Noldus Information Technology, VA).

### Brain sample collection

On day 8 post infection, mice were euthanized with halothane to collect hippocampi. Hippocampi were collected into CentriStar™ cap 15 ml Corning® centrifuge tubes (Corning, NY) in Isove’s Modified Dulbecco’s Medium (IMDM) (GIBCO/Invitrogen; Carlsbad, CA), 10% Fetal Calf Serum (FCS), and penicillin streptomycin (P/S) on ice. Tissue was pushed through 40 μm nylon cell strainers (Falcon®, Corning Incorporated, NY), centrifuged at 1200 rmp at 4 °C for 10 min and re-suspended in 450 μl IMDM buffer for flow cytometry staining.

### FACS of Hippocampal isolates

Flow cytometry was used to determine macrophage and microglia populations in single cell preparations of the hippocampus prepared in complete media; IMDM (GIBCO/Invitrogen; Carlsbad, CA), 10% FCS, and P/S on ice. Samples were stained with an antibody mix (MACS buffer +1% inactivated Rat serum, 1% α-FcγII/III (clone 2.4G2), α-F4/80 (clone GK1.5; BD Pharmingen^TM^, FITC), and α-Iba1 (clone GR106078-1; Abcam®, FITC) for 30 min on ice, and then fixed in 2% paraformaldehyde before permeabilization (0.5 g saponin, 0.055 g CaCl2, 0.0625 g MgSO4, 0.25 g NaN3, 0.5 g BSA, 10 mM HEPES in final volume of 500 ml of 1X PBS) for 1 hour at 4 °C. Intracellular staining of samples was done with an antibody mix (MACS buffer +2% inactivated Rat serum, 2% α-FcγII/III (clone 2.4G2), α-IL-1α (clone ALF-161; BioLegend®, PE); α-IL-6 (Lot 99523; BP Pharmingen^TM^, PE); α-iNOS2 (clone Sc651; Santa Cruz Biotechnology®), α-MHCII (clone M5/114.15.2; eBioscience®, AlexaFluor 700), α-IL-4 (clone 11B11; eBioscience®, APC); α-IL-13 (clone eBio13A; eBioscience®, PE Cy7); and α-Arginase1 (clone H1010; Santa Cruz Biotechnology®) for 30 min on ice and read by a Becton Dickinson FACS FORTESSA machine (BD San Diego, CA). Data was analyzed by FlowJo© Treestar (Ashland, OR) and graphed with GraphPad Prism® software. Unless otherwise stated, antibodies were from BD Pharmingen^TM^.

### Statistical Significance

Statistical significance was measured by two-tailed unpaired student t-tests, or two-way analysis of variance (ANOVA) corrected for multiple comparisons with a Bonferroni post-hoc. GraphPad Prism v 6.0 was used for analyses, with a ‘p’ value of less than 0.05 considered significant (*p < 0.05, **p < 0.01, ***p < 0.001).
